# *Uvariopsis dicaprio* (Annonaceae) a new tree species with notes on its pollination biology, and the Critically Endangered narrowly endemic plant species of the Ebo Forest, Cameroon

**DOI:** 10.7717/peerj.12614

**Published:** 2022-01-06

**Authors:** George Gosline, Martin Cheek, Jean Michel Onana, Eric Ngansop Tchatchouang, Xander M. van der Burgt, Lorna MacKinnon, Léo-Paul M. J. Dagallier

**Affiliations:** 1 Science, Royal Botanic Gardens, Kew, Richmond, Surrey, United Kingdom; 2 Faculty of Science, Department of Plant Biology, University of Yaoundé I, Yaoundé, Cameroon; 3 National Herbarium of Cameroon, IRAD, Yaoundé, Cameroon; 4 DIADE, Univ Montpellier, CIRAD, IRD, Montpellier, France

**Keywords:** Cauliflorous, Conservation, Cross-sanaga interval, Moth-pollination, Threatened species

## Abstract

**Background:**

The Ebo Forest area is a highly threatened centre of diversity in the Littoral Region of Cameroon, globally important for conservation with many threatened species including 68 threatened species of plant, yet not formally protected. The tropical African evergreen forest tree genus *Uvariopsis* Engl. & Diels (Annonaceae) is characterised by unisexual, usually cauliflorous flowers with a uniseriate corolla of four petals, and two sepals. Cameroon is the centre of diversity of the genus with 14 of the 19 known species.

**Methods:**

The herbarium collection *MacKinnon* 51 from Ebo is hypothesized to represent a new species to science of *Uvariopsis*. This hypothesis is tested by the study of herbarium specimens from a number of herbaria known to hold important collections from Cameroon and surrounding countries.

**Results:**

We test the hypothesis that *MacKinnon* 51 represents a new species to science, using the most recent dichotomous identification key, and comparing it morphologically with reference material of all known species of the genus. We make a detailed comparative morphological study focussing on three other Cameroonian species, *Uvariopsis solheidii, U. korupensis* and the sympatric *U. submontana*. In the context of a review of the pollination biology of *Uvariopsis*, we speculate that in a genus otherwise with species with dull, flesh-coloured (pink, red to brown) flowers pollinated (where known) by diptera, orthoptera and blattodea (flies, crickets and cockroaches), the glossy, pale yellow-green flowers of *Uvariopsis dicaprio*, with additional traits unique in the genus, may be pollinated by nocturnal moths. Based on *MacKinnon* 51, we formally name *Uvariopsis dicaprio* Cheek & Gosline (Annonaceae) as new to science, and we describe, and illustrate, and map it. Restricted so far to a single site in evergreen forest in the Ebo Forest, Littoral Region, Cameroon, *Uvariopsis dicaprio* is provisionally assessed as Critically Endangered using the IUCN, 2012 standard because the forest habitat of this species remains unprotected, and there exist imminent threats of logging and conversion to plantations.

**Discussion:**

We show that the highest density of species of the genus (12), and of narrow endemics (5), is found in the Cross-Sanaga Interval of SE Nigeria and Western Cameroon. A revised key to the 14 Cameroonian species of *Uvariopsis* is presented. We review the other seven narrowly endemic and threatened species unique to the Ebo forest of Cameroon and discuss the phytogeographic affinities of the area.

**Conclusions:**

*Uvariopsis dicaprio* adds to the growing list of species threatened with extinction at Ebo Forest due to current anthropogenic pressures.

## Introduction

A long-running survey of plants in Cameroon to support improved conservation management has been in course since 1992. The survey is led by botanists from the Royal Botanic Gardens, Kew and IRAD (Institute of Agricultural Research for Development)-National Herbarium of Cameroon, Yaoundé. The study has focussed on the Cross-Sanaga interval (Cheek et al., 2001, 2006) which contains the area with the highest plant species and generic diversity per degree square in tropical Africa (Barthlott, Lauer & Placke, 1996; Dagallier et al., 2020). The herbarium specimens collected in these surveys formed the foundations for a series of Conservation Checklists (see below). So far, over 100 new species and several new genera have been discovered and published, new protected areas have been recognised and the results of analysis are feeding into the Cameroon Important Plant Area programme (https://www.kew.org/science/our-science/projects/tropical-important-plant-areas-cameroon), based on the categories and criteria of Darbyshire et al. (2017).

**Figure 1:**
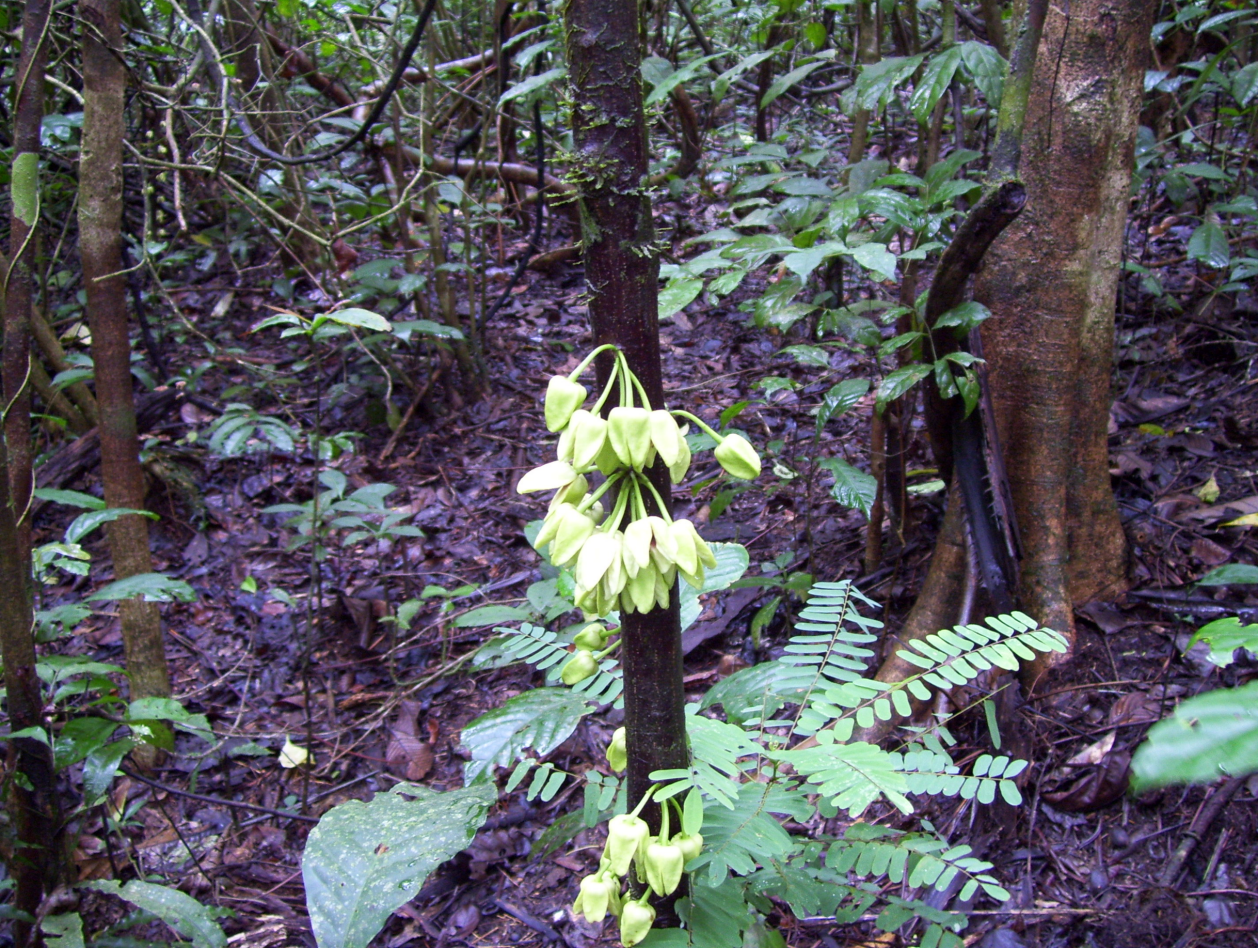
*Uvariopsis dicaprio*. Cauliflorous inflorescences on trunk. Photo Lorna MacKinnon.

**Figure 2:**
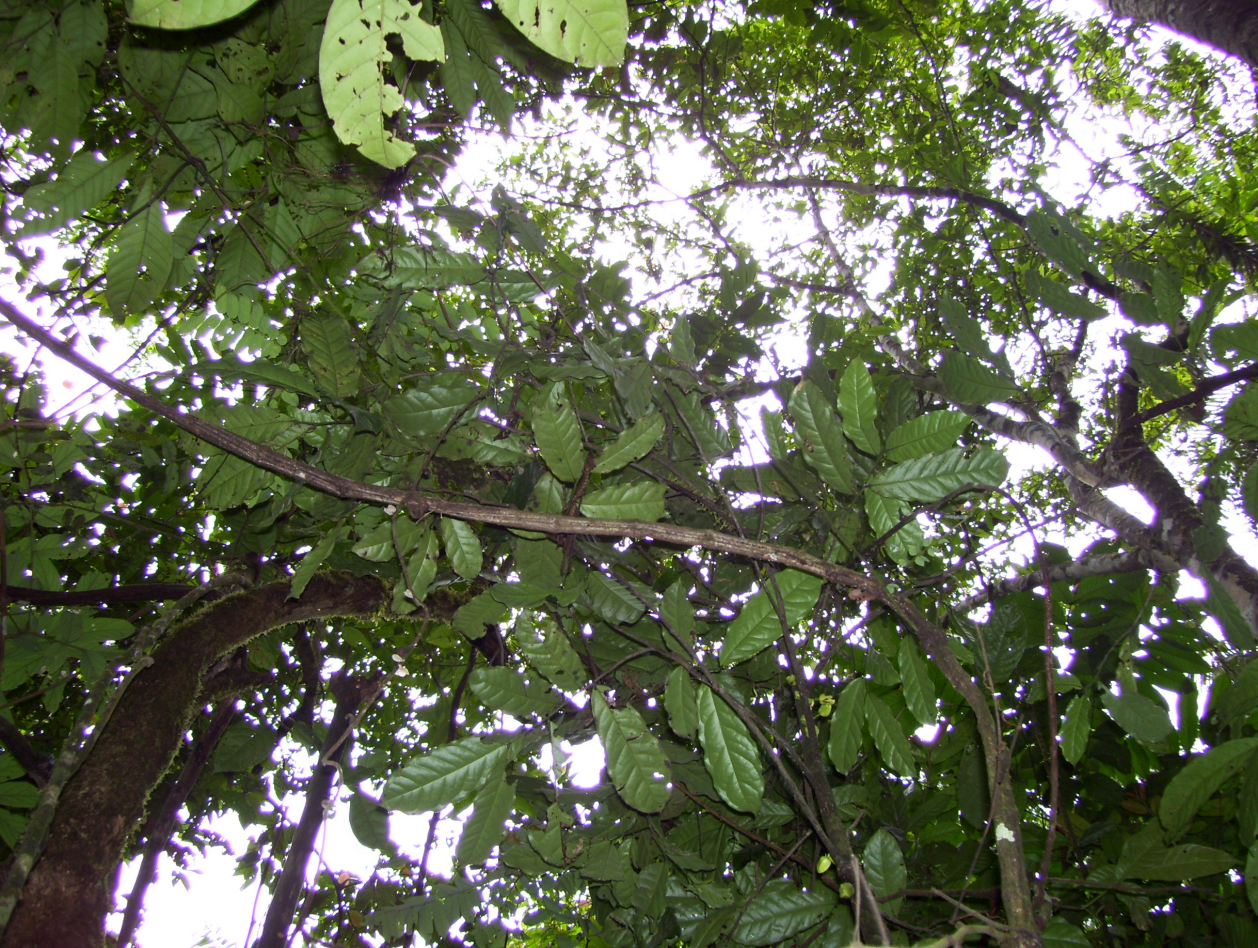
*Uvariopsis dicaprio*. Trunk apex with cauliflorous flowers and canopy. Photo Lorna MacKinnon.

**Figure 3:**
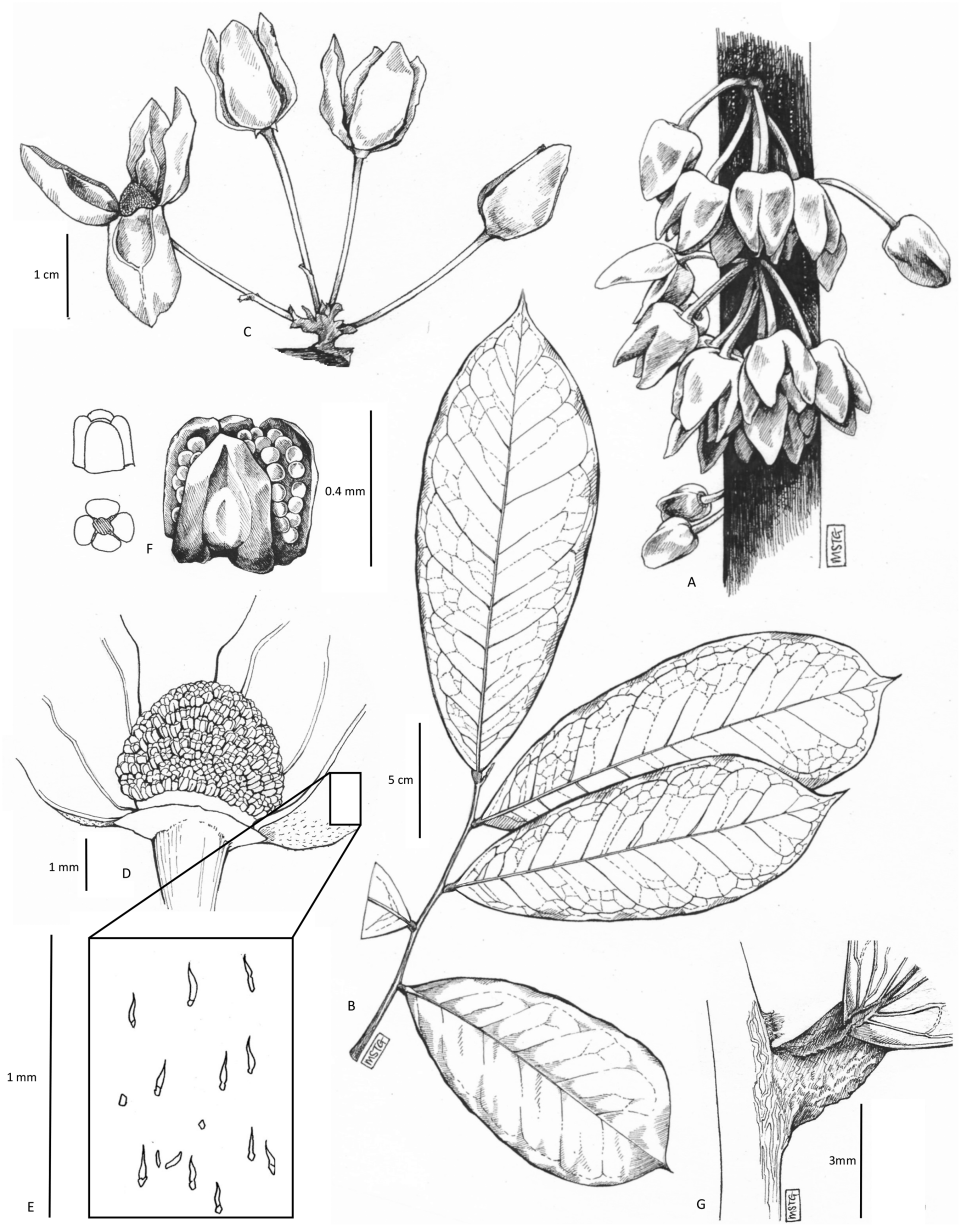
*Uvariopsis dicaprio*. (A) habit, cauliflorous inflorescences on trunk; (B) leafy branch, one season’s growth; (C) inflorescence, showing pedicel articulations, bracts and bracteoles; (D) flower, with one petal removed to show the staminal dome; (E) detail of sparse hairs on abaxial petal surface; (F) stamen, different views; (G) junction of base of leaf with stem, showing dome-like axillary bud. All drawn from *MacKinnon* 51 (K) by MEG GRIFFITHS.

In connection with preparation of a Conservation Checklist of the plants of the Ebo Forest, Littoral Region, a plant specimen (*MacKinnon* 51, Figs. 1–3) was identified as an *Uvariopsis* Engl. which resembled no other species known in the genus. In this paper we test the hypothesis that it is a new species to science endemic to Ebo and name the new species as *Uvariopsis dicaprio*. We also review other endemic and near-endemic plant species of the Ebo Forest (see “Discussion”).

*Uvariopsis* (Annonaceae, subfamily Annonoideae, tribe Monodoreae) (Chatrou et al., 2012; Guo et al., 2017) is a highly distinctive and easily recognised genus, since most of its species have unisexual flowers, a calyx with two basally connate sepals, and the petals in a single whorl of four (very rarely three, see below). Annonaceae are otherwise characterised by bisexual flowers with trimerous perianths (van Heusden, 1992). Most species of the genus *Uvariopsis* are cauliflorous small trees, the flowers being produced from the trunk, although some species are ramiflorous or bear axillary flowers (Kenfack et al., 2003; Couvreur et al., in press).

Nineteen species are currently accepted in *Uvariopsis*. Five species have been published in the 21^st^ Century: *Uvariopsis korupensis* Gereau & Kenfack (2000), *U. submontana* Kenfack et al. (2003) and *U. etugiana* Couvreur et al. (in press) all from Cameroon, *U. citrata* Couvreur & Niangadouma (2016) from Gabon and *U. lovettiana* Couvreur & Luke (2010) from Tanzania. In addition a sixth species, *Uvariopsis tripetala* (Baker f.) G.E.Schatz, was transferred to the genus from the monotypic *Dennettia* Baker f. (Kenfack et al., 2003), although it is likely that *Dennettia* should be reinstated (Cheek, Luke & Gosline, 2021). The genus is centred in Cameroon, where 13 of the 19 species occur, followed in species diversity by Gabon, with six species (Sosef et al., 2006; Couvreur & Niangadouma, 2016). The most widespread species of the genus is *Uvariopsis congensis* Robyns & Ghesq. which occurs from Cameroon to South Sudan, Zambia and Kenya. Several species are rare, being known from only one or two specimens and have restricted ranges, these include *U. etugiana* (Cameroon endemic) and *U. citrata* (Cameroon & Gabon), both known from two specimens, and *U. sessiliflora* (Mildbr. & Diels) Robyns & Ghesq. endemic to Cameroon and known from a single specimen.

The genus is distributed throughout continental tropical African evergreen forests, from Guinea in the West to Tanzania in the east, and as far south as northern Zambia. The species usually occur at low altitude, exceptions including *U. submontana* and *U. lovettiana* which occur in submontane or cloud forest in Cameroon and Tanzania respectively (Kenfack et al., 2003; Couvreur & Luke, 2010). Species are usually small trees in high quality, undisturbed forest and appear not to be pioneers. They usually occur at low frequency. For example, in the Mefou Proposed National Park of Central Region Cameroon, only a single mature individual with one juvenile of one species of the genus, *U. solheidii* (De Wild.) Robyns & Ghesq., was found in the course of many weeks of botanical surveys by numerous botanists collecting thousands of specimens (Cheek, Harvey & Onana, 2011). However, in rare ecological circumstances, some species can become locally dominant *e.g*., *U. tripetala* (*Dennettia tripetala*) in the understorey of maritime lowland evergreen inselberg forest in Guinea (Couch et al., 2019), and also, *U. congensis* locally subdominant in forests in western Uganda where it flowers synchronously and is dispersed by primates (Dominy & Duncan, 2005; G. Gosline, 2016, personal observation).

## Materials & Methods

*The electronic version of this article in Portable Document Format (PDF) will represent a published work according to the International Code of Nomenclature for algae, fungi, and plants (ICN), and hence the new names contained in the electronic version are effectively published under that Code from the electronic edition alone. In addition, new names contained in this work which have been issued with identifiers by IPNI will eventually be made available to the Global Names Index. The IPNI LSIDs can be resolved and the associated information viewed through any standard web browser by appending the LSID contained in this publication to the prefix “*http://ipni.org/*”. The online version of this work is archived and available from the following digital repositories: PeerJ, PubMed Central, and CLOCKSS*.

Fieldwork and specimen collection was as previously described in Cheek, Onana & Chapman (2021), under the same system of agreements, permits and approvals. The herbarium specimen was made using the Schweinfurth method, that is, initially preserved in ethanol until it could be dried in the standard way (Bridson & Forman, 1998) The first duplicate was deposited at YA, the remainder sent to K for identification and distribution following standard practice.

Herbarium citations follow Index Herbariorum (Thiers, in press). Specimens were viewed at EA, K, P, WAG, and YA. The National Herbarium of Cameroon, YA, was searched for additional material of the new species, but without success. Images for specimens at WAG were studied at https://bioportal.naturalis.nl/?language=en and those from P at https://science.mnhn.fr/institution/mnhn/collection/p/item/search/form?lang=en_US. We also searched JSTOR Global Plants (https://plants.jstor.org/ accessed March 2021) for additional material, and finally the Global Biodiversity Facility (GBIF, www.gbif.org accessed March 2021). We compared our material with reference material of all other species in the genus. Binomial authorities follow the International Plant Names Index (IPNI 2021). The conservation assessment was made using the categories and criteria of IUCN (2012), adopting the 4 km^2^ cell-size preferred by IUCN. Since a single site is known for the species, it was not feasible to calculate a convex polygon for the extent of occurrence. Herbarium material was examined with a Leica Wild M8 dissecting binocular microscope fitted with an eyepiece graticule measuring in units of 0.025 mm at maximum magnification. The drawing was made with the same equipment using Leica 308700 camera lucida attachment. The botanical terms follow Beentje & Cheek (2003), and format of the description follow the conventions of Kenfack et al. (2003) and Couvreur & Luke (2010). The map was made using QGIS 3.12 (https://www.qgis.org).

## Results

**Comparative morphology**

The new *Uvariopsis dicaprio* has leaves exceeding 15 cm long, cauliflorous flowers with pedicels exceeding 10 mm long, petals free, exceeding 7 mm long, which grouped it with the other species of *Uvariopsis* in Cameroon (Couvreur et al., in press). *Uvariopsis dicaprio* is similar to *U. solheidii* by the conical flower buds that in *U. dicaprio* can vary from ovoid-conical to pyramidal. The two species can be separated using the differential characters in Table 1 below.

**Table 1:** Differential characters separating *Uvariopsis dicaprio, U solheidii, U. korupensis* and *U.submontana*. Data for *Uvariopsis solheidii* taken mainly from Couvreur et al. (in press) and Cheek, Harvey & Onana (2011), for *Uvariopsis korupensis Gereau & Kenfack (2000)*, and for *Uvariopsis submontana* (Kenfack et al., 2003).

Character	*Uvariopsis solheidii*	*Uvariopsis dicaprio*	*Uvariopsis korupensis*	*Uvariopsis submontana*
Indumentum of stem, petiole and abaxial midrib	Tomentose	Glabrous	Appressed pubescent	Appressed pubescent
Leaf-blade dimensions (cm)	16.6–29 × 5–9.5	17.7–20.3 (–23) × (6.4–) 7–7.9	30–52 × 9–14	6–38 × 5–11
Number of secondary nerves on each side of midrib	8–13	5–8 (–9)	13–20	9–18
Number of flowers per inflorescence	1–3	(1 –)4–7	2–3	6–50
Flower shape (mature bud)	Ovoid-conic	Ovoid-conic to pyramidal	Ovoid-conic to pyramidal	Ovoid-conic to pyramidal
Petal colour	Wine brown	Yellow-green	Pink-purple	Pink-Purple
Petal dimensions (mm)	7–10 × 2.5–5	(14–)16 × (5.5–) 9	10–25 × 5–10	7–15 × 5–7
Petal texture	Thick, fleshy	Thin, leathery	Thick, fleshy	Thick, fleshy
Inner surface petals	Tuberculate?	Smooth	Tuberculate	Tuberculate
Outer surface petals	Appressed-pubescent	Mostly naked, hairs few, widely scattered	Appressed-pubescent	Appressed-pubescent
Petals at base	Free	Free	United	United

However, based on morphology, *Uvariopsis dicaprio* is closely similar to two other Cameroonian species, *U. korupensis* and *U. submontana*. These four species, including *U. solheidii*, share the following features: they are all cauliflorous with well-developed (1.5–8 cm or more long) pedicels, flowers which in bud are ovoid-conic, and more-or-less pyramidal (most strongly so in *Uvariopsis dicaprio* where the angles of the pyramid become wing-like: Fig. 1), petal shapes are more or less ovate-lanceolate, rather than orbicular as in most species of the genus. The four species can be compared and separated from each other using the characters in Table 1.

The petals of *Uvariopsis dicaprio* do not seem to reflex at anthesis exposing the staminal dome as they do in the other species, rather they open only slightly, concealing the staminal dome (Fig. 1). Moreover, the petals are only thinly leathery in texture, with an inner surface that is smooth, and not thick and fleshy with a tuberculate inner surface as in the other three species. Another difference is that the outer surface of the petals is mostly naked, with only a very few widely scattered hairs, not appressed-pubescent as in the other species (Fig. 3, Table 1). In leaf-blade dimensions *Uvariopsis dicaprio* fits within the ranges of those of most of the other species, and also in petal dimensions where it fits closest to the ranges of *Uvariopsis korupensis* and *Uvariopsis submontana*. Vegetatively *Uvariopsis dicaprio* differs from all three other species in having a much lower range of secondary nerves (5 to 8 (–9) *versus* 8 to 20), and can immediately be distinguished from them by having glabrous young stems, petioles and abaxial midribs (*versus* appressed pubescent or tomentose) (Table 1).

*Uvariopsis dicaprio* additionally has several features that appear unique in the genus that are presented in the notes section following the description below.

The new species can be separated from all other Cameroonian species of *Uvariopsis* using the key presented below, modified from Dagallier in Couvreur et al. (in press).

### Key to the species of *Uvariopsis* (and *Dennettia*) in Cameroon

1. - Crushed leaves emitting a strong citrus scent*U. citrata*
- Crushed leaves without citrus scent2
2. - Leaf blades 7.2 – 15.5( – 18) cm long; pedicel 0 – 7( – 9) mm long3
- Leaf blades (11.1 – )16.3 – 38( – 61.5) cm long, pedicel (3 – )8 – 60( – 450)mm long 6
3. - Flowers cauliflorous, pedicel 0 – 2 mm long*U. sessiliflora *
- Flowers ramiflorous, pedicel (0 – 2)3 – 11 mm long, sometimes cauliflorous4
4. - Flowers bisexual, petals 3 ( – 4)*Dennettia tripetala*
- Flowers unisexual, petals 45
5. - Young branches glabrous or very sparsely pubescent, petals free*U. congensis*
- Young branches densely to sparsely pubescent, petals basally fused *U. zenkeri*
6. - Petals basally fused7
- Petals free10
7. - Petals 3*U. congolana*
- Petals 48
8. - Flower buds globose, monocarps verrucose*U. pedunculosa*
- Flower buds conical to pyramidal, monocarps smooth9
9. - Sepals 5 – 10 mm long, flowers completely covering base of trunk, generally occurring above 800 m a.s.l.*U. submontana*
- Sepals 2 – 4 mm long, flowers partially covering base of trunk, generally occurring below 800 m a.s.l.*U. korupensis*
10. - Flowering pedicels 3 – 8 mm long11
- Flowering pedicels 10 – 160 mm long12
11. - Petals linear, 25 – 45 mm long, more than 6 times longer than wide*U. bakeriana*
- Petals elliptic to ovate, 10 – 14 mm long, less than 6 times longer than wide*U. etugiana*
12. - Flower buds globose*U. dioica*
- Flower buds conical or narrowly ovoid to pyramidal13
13. - Stems and petioles tomentose, lateral nerves 8 – 13 on each side of the midrib; flowers wine brown*U. solheidii*
- Stems and petioles glabrous; lateral nerves 5 – 8( – 9) on each side of the midrib; flowers green-yellow*U. dicaprio*

***Uvariopsis dicaprio*** *Cheek & Gosline* **sp. nov.** Type: Cameroon, Littoral Region, Yabassi, Ebo Forest, 4° 20′ 44″ N, 10° 24′ 33″ E, 849 m alt. Dicam Trail 2000 m from Bekob camp, male fl. 25 March 2008, *MacKinnon* 51 (holotype K001381842; isotypes MO, YA).

Syn. *Uvariopsis ebo nom. nud*. (Gosline et al., 2021: 5).

Diagnosis. Similar to *Uvariopsis solheidii* (De Wild.) Robyns & Ghesq., differing in the stem, petioles and abaxial midrib glabrous (*versus* tomentose); number of secondary nerves on each side of the midrib 5–8 (*versus* 8–13); petals yellow-green, (14–) 16 × (5.5–) 9 mm (*versus* wine brown, 7–10 × 2.5–5 mm).

*Cauliflorous, probably monoecious understorey tree* 3–4 m tall. Trunk terete, lacking flutes or prop roots, 1.8–2.5 cm diameter at 1.5 m above the ground, bark smooth, dark-brown, with sparse, longitudinal lines of white lenticels (Fig. 1), the crown sparsely branched (Fig. 2). Leafy stems with 3–4 leaves per season’s growth, terete, internodes (1.2–) 1.5–2.8 (–4.3) cm long, 0.15–0.2 cm diam., pale yellow-green, later orangish brown, glabrous. Axillary buds dome-shaped 0.5–0.75 × 1 mm, bud-scales numerous, linear, spreading, densely hairy, hairs simple, appressed, c. 0.5 mm long, colourless or red brown. *Leaves* distichous, with punctations (minute translucent glands in the interior of the blade), lacking scent when crushed (collection metadata, *MacKinnon* 51), blades oblanceolate 17.7–20.2 (–23) × (6.4–) 7–7.9 cm, acumen narrowly triangular (0.5–) 1–1.3 cm long, base broadly acute with convex edges, minutely cordate, blade mounted above petiole, margins undulate-sinuous (live & dried), midrib impressed on adaxial surface, inconspicuous, below a groove; on abaxial surface subcylindrical, 1–1.2 mm diam., conspicuous; secondary veins 5–8 (–9) on each side of the midrib, brochidodromous, arising at c. 50° from the midrib, initially straight, then curving in the outer third, uniting with the secondary nerve above to form a looping inframarginal nerve, attaining 3–4 mm from the margin; intersecondary nerves sometimes present, tertiary nerves raised, conspicuous, forming a reticulum with cells 4–5 mm long, quaternary nerves inconspicuous; glabrous (except in bud when densely orange-brown hairy, hairs c. 0.1 mm long). Petiole stout, shallowly canaliculate, c. 4 (–5) mm long, 1.9–2.1 mm diam., narrowing at base and apex, adaxial groove shallow, c. 0.5 mm wide, glabrous. *Female inflorescences* unknown. *Male inflorescences* cauliflorous, scattered along the trunk from near ground level to the top of the trunk 2.5–3 m above the ground, each 1–7-flowered (Figs. 1 & 2). Peduncles patent, c. 2 × 2 mm, pale brown, glabrous, bearing sub-umbellate, radiating, 1-flowered partial-peduncles. Partial-peduncles 0.5–2 × 0.9–1.2 mm, terminating in 1–2 bracts subtending a pedicel. Bracts oblong-elliptic, 1.5 × 0.5–0.6 mm, apex acute, outer surface about 50% covered in appressed white hairs c. 0.15–0.2 mm long, inner surface glabrous. *Male flowers*. Pedicels 1.8–2.5 cm long, 0.1 cm diam., articulated with the partial-peduncle, with (0–) 1 (–2) scattered, bracteoles in the proximal few mm. Bracteoles similar to the bracts, ovate-oblong, shortly sheathing, (1–) 1.25 × 1 mm, outer surface with sparse scattered simple appressed translucent hairs 0.05–0.2 mm long, buds narrowly ovoid to pyramidal, c.16 × 11 mm. *Sepals* 2, opposite, drying pale brown, reflexed, semi-orbicular, 1–1.5 × 2.1–2.5 mm, glabrous. *Petals* 4, uniseriate, free, thinly leathery, pale, glossy, yellow-green when live (Fig. 1), drying black, lanceolate-oblong, (14–) 16 × (5.5–) 9 mm, not fleshy but c. 0.25–0.3 mm thick, apex rounded, base rounded, outer surface sparsely and inconspicuously hairy, hairs 7–9 per mm^2^ (5% of surface covered), simple, translucent, appressed, c. 0.1 mm long, apices rounded. Inner surface of petals smooth, non-tuberculate, with a shallow elliptic-oblong excavation c. 8 × 5 mm, the margin of the excavation raised, the apex with a ridge extending along the midline to the petal apex, glabrous apart from a few scattered erect, minute white hairs 0.05 mm long at the excavation apex. Staminal dome 3.5–4 mm long, 3.5–4 mm diam., consisting of stamens and a receptacular torus. Stamens shortly cylindrical-angular, c. 0.5 × 0.1 (–0.2) mm, connective with two lateral extrorse longitudinal anther cells, each exceeding the connective. Apical connective appendage absent. Female flowers, fruit and seed unknown. Figs. 1–3.

**Figure 4:**
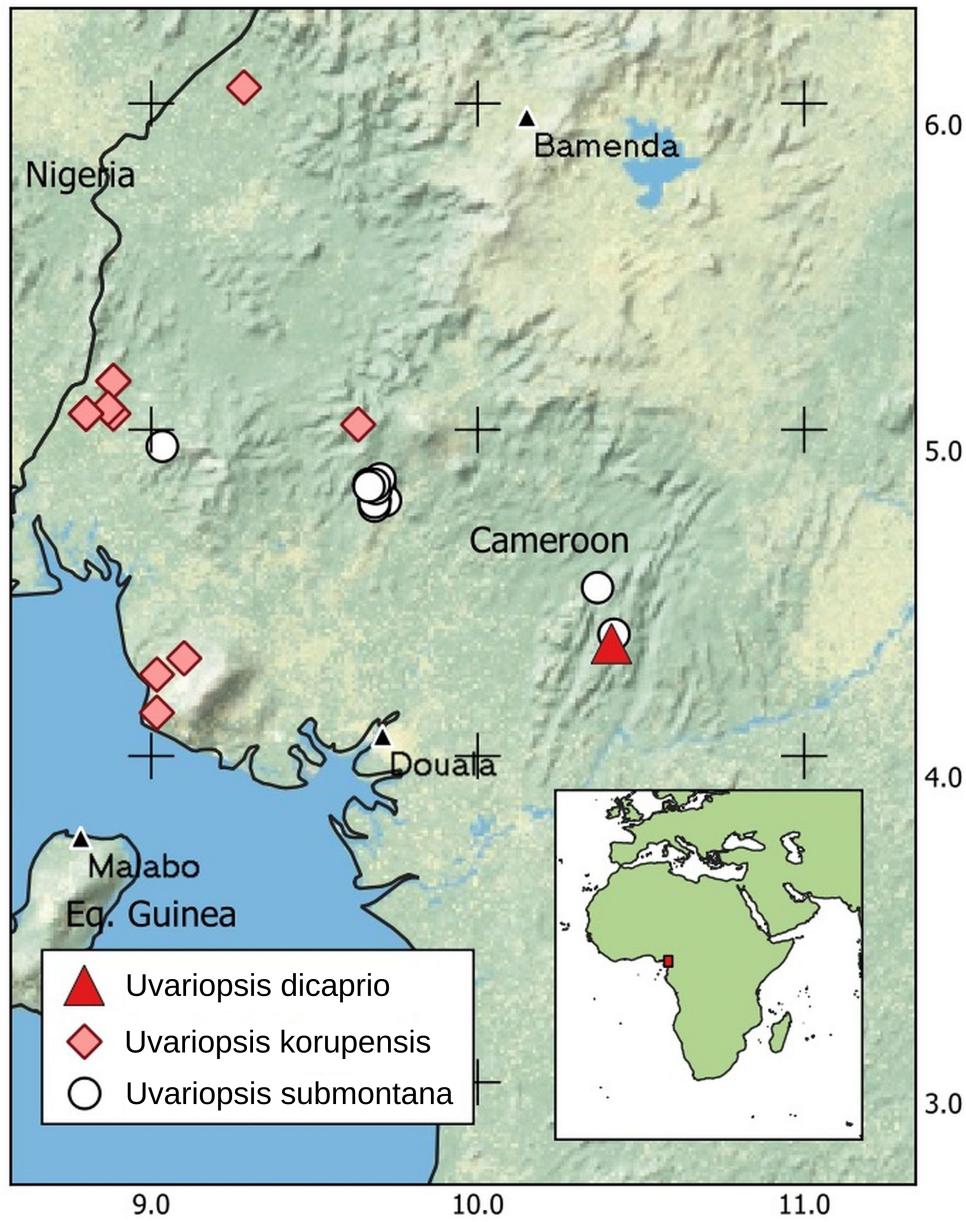
Global distribution of *Uvariopsis dicaprio*, together with *U. korupensis* and *U. submontana*.

**DISTRIBUTION.** Cameroon (Fig. 4) endemic to the Ebo Forest of the Littoral Region on present evidence.

**HABITAT.** *Uvariopsis dicaprio* is so far only known from lower submontane forest (850 m elev.). below the elevation for the upper montane forest indicator species *Podocarpus latifolius* (Thunb.) R.Br. ex Mirb. The geology is ancient, highly weathered basement complex, with some ferralitic areas in foothill areas which are inland, c. 100 km from the coast. Altitude varies from c. 200 m to 1,200 m elevation. The wet season (successive months with cumulative rainfall >100 mm) falls between March and November and is colder than the dry season. Average annual rainfall at Bekob measured 2010–2016 is 2,336 mm (E. Abwe, 2018 Ebo Forest Research Programme, Cameroon, personal communication, Abwe & Morgan, 2008; Cheek et al., 2018a).

**CONSERVATION STATUS.** *Uvariopsis dicaprio* is currently known from a single specimen with all male flowers at a single location inside the mid-eastern part of the Ebo Forest (Fig. 4). Less than 50 mature individuals have been observed (B. Morgan, 2021, personal communication), despite the species being highly conspicuous in flower (Fig. 1) and situated on a major footpath close to a research camp used by many biologists over the last 15 years.

Since 2006, botanical surveys have been mounted almost annually, at different seasons, over many parts of the formerly proposed National Park of Ebo. About 2,500 botanical herbarium specimens have been collected, but this species has not yet been seen elsewhere in the c. 2,000 km^2^ of the Ebo Forest. However, the area outside the two research camps, especially the western edge, has not been fully surveyed for plants. While it is likely that the species will be found at additional sites within the Ebo Forest, there is no doubt that it is genuinely range-restricted as are some other species of *Uvariopsis* in Cameroon (see “Introduction”). Botanical surveys and other plant studies for conservation management in forest areas north, west and east of Ebo resulting in tens of thousands of specimens being collected and identified have failed to find any additional specimens of this species (Cheek et al., 1996; Cable & Cheek, 1998; Cheek, Onana & Pollard, 2000; Maisels, Cheek & Wild, 2000, Harvey et al., 2004; Cheek et al., 2004; Cheek, Harvey & Onana, 2010; Harvey, Tchiengue & Cheek, 2010; Cheek, Harvey & Onana, 2011).

The area of occupation of *Uvariopsis dicaprio* is estimated as 4 km^2^ using the IUCN preferred cell-size. The extent of occurrence is the same. In February 2020 it was discovered that moves were in place to convert the forest into two logging concessions (*e.g*. https://www.globalwildlife.org/blog/ebo-forest-a-stronghold-for-cameroons-wildlife/ and https://blog.resourceshark.com/cameroon-approves-logging-concession-that-will-destroy-ebo-forest-gorilla-habitat/ both accessed 12 April 2021). Such logging would result in timber extraction that would open up the canopy and remove the intact habitat in which *Uvariopsis dicaprio* is found. Additionally, slash and burn agriculture often follows logging trails and would negatively impact the population of this species. Fortunately the logging concession was suspended in August 2020 due to representations to the President of Cameroon on the global importance of the biodiversity of Ebo (https://www.businesswire.com/news/home/20200817005135/en/Relief-in-the-Forest-Cameroonian-Government-Backtracks-on-the-Ebo-Forest accessed 12 April 2021). However, the forest habitat of this species remains unprotected and threats of logging and conversion of the habitat to plantations remain, and mining is also a threat. *Uvariopsis dicaprio* is therefore here assessed as Critically Endangered, CR B1+2ab(iii), D.

**PHENOLOGY.** Flowering has been observed in late March and early April (B. Morgan, 2021, personal communication).

**ETYMOLOGY.** This threatened and spectacular tree is named for the American actor and conservationist Leonardo DiCaprio, who, through several months in 2020, lobbied extensively on social media (*e.g*. https://www.instagram.com/p/B_0LSAhFRue/?hl=en; https://twitter.com/leodicaprio/status/1257729388314943490?lang=en both accessed 12 April 2021) to draw attention to threats for the numerous rare Ebo species from the logging concession that had been announced at Ebo earlier that year. The concession was cancelled in August 2020, surely partly due to his efforts.

**VERNACULAR NAMES & USES.** None are known.

**NOTES.** The distal half of the petals and the margins of the proximal half are flat, wing-like and held against each other (applanate) in bud. In section therefore, the distal part of the corolla will appear cross-shaped (see Fig. 1). This seems to be an extreme form of the petal structure and pyramidal flower bud shape seen in the probably closely related Cameroonian species *Uvariopsis korupenis* and *U. submontana* (see results, above). *Uvariopsis dicaprio* is further distinct from all other species of the genus in that a distinct peduncle is present that bears several branches (partial-peduncles) each of which bears and is articulated with a single pedicel (Fig. 3). Other cauliflorous species of *Uvariopsis* have few to many-flowered fasciculate inflorescences, a peduncle not being observed, the pedicels arising directly from a perennial woody burr. In non-cauliflorous species of *Uvariopsis* the inflorescences consist of a single, axillary flower.

It was first intended to name *Uvariopsis dicaprio* as *U. ebo* and this name was used in the bioRxiv pre-print (Gosline et al., 2021: 5). However, such pre-prints have no standing as publications for nomenclatural purposes according to the Code (Turland et al., 2018) because they not intended by the authors as the final publication. Therefore *Uvariopsis ebo* is classed as a *nominum nudum*.

## Discussion

### Pollination biology in *Uvariopsis* and *Uvariopsis dicaprio*

Unusual and distinctive features of *Uvariopsis dicaprio* within the genus include the colour, shape and texture of the corolla. Bright, glossy, pale yellow-green petals are otherwise unknown in a genus where the petals are otherwise dull shades of pink to purple and brown. Unlike in all other species of the genus known, the petals are not thick and fleshy but thinly leathery. The centre of the proximal half of each petal is concave before anthesis, forming a globose chamber for the staminal dome with the other three petals (Fig. 1). On the inner surface of the petal, the concave area is demarcated by an inverted U-shaped, distinct, raised, broad ridge which seems to be the point of contact with those of the other sepals, sealing the chamber. Such a structure has not been reported or observed in other species of the genus.

A striking feature of *Uvariopsis dicaprio* is the presentation and colour of the flowers. Of the 14 species of the genus in Cameroon, 11 are cauliflorous. All of these except *U. dicaprio* have petals which are shades of pink, red, purple to brown. Cauliflorous *Uvariopsis* species present their flowers more or less perpendicular to the trunk; the flowers of *U. dicaprio* are pendant and with the corolla opening only slightly (see “Results”), and facing the ground (Fig. 1). All species of *Uvariopsis* other than *U. bisexualis* Verdc. are monoecious. The majority of Annonaceae species are protogynous hermaphrodites, most often beetle pollinated, some exhibiting thermogenesis (Gottsberger, 2012). In the monoecious *U. bakeriana* (Hutch. & Dalziel) Robyns & Ghesq. and *U. congolana* (De Wild.) R.E.Fr. the female flowers mature before the male (Gottsberger, Meinke & Porembski, 2011), and our evidence for *U. dicaprio* suggests the same sequence. In *U. submontana, U. korupensis, U. congolana, U. dioica*, and *U. pedunculosa*, the male flowers are higher on the trunk, with the female flowers clustered towards the base of the trunk. In *U. dioica* and *U. korupensis* the female pedicels are generally less than 10 cm long and in *U. korupensis* the fleshy fruits mature in dense concentrations at the base of the trunk where they can attract ground-dwelling animals.

In *Uvariopsis congolana*, (Diels) Robyns & Ghesq. and *U. pedunculosa* (Diels) Robyns & Ghesq. the female globular flowers are born on slender pedicels to c. 50 cm long embedded in the leaf litter. This habit is also exhibited by *Isolona cauliflora* Verdc. Gottsberger, Meinke & Porembski (2011) studied the visitors to *U. pedunculosa* (identified as *U. congolana*) and concluded that pollination likely was by small litter-flies (not beetles). “It is suspected that this predominantly sapromyiophilous species has a pollination system mimicking fungi and/or carcass, and that the pollinating flies normally live on fungi and/or carcass.” They also conclude that *U. bakeriana* is pollinated by dung-flies. Mertens et al. (2018) studied *U. dioica* and found minimal visitation by Lepidoptera but more by nocturnal crickets and cockroaches. In all three species of *Uvariopsis* where pollination studies have been done, the researchers have described the pollinators as scarce and unpredictable, and thermogenesis has not been observed.

In this context, it seems likely that *U. dicaprio* has found a different pollinator from its sister taxa. The bright pale yellow-green flowers suggest nocturnal pollinators. The similarly glossy, yellow-green flowers of the Asian, widely cultivated ylang-ylang, *Cananga odorata* (Lam.) Hook.f. & Thomson are pollinated by nocturnal moths and small beetles (Parrotta, 2014). We speculate that the flowers of *Uvariopsis dicaprio*, unique in the genus, may also be adaptated for moth pollination, otherwise unrecorded in indigenous African Annonacaeae (Gottsberger, 2012). Concerted field monitoring to observe pollinators is needed during the flowering season of *Uvariopsis dicaprio* to test this hypothesis.

### The centre of diversity of *Uvariopsis*

With the recognition in this paper of *Uvariopsis dicaprio*, 20 species are now accepted in *Uvariopsis* (see introduction). The highest species diversity for any country is found in Cameroon, now with 14 species, and with six of these nationally endemic. However, the biogeographic area with highest species diversity is the much smaller area of the Cross-Sanaga Interval which includes 12 species, of which five are globally endemic including *Uvariopsis dicaprio*. The Cross-Sanaga Interval appears to be the main centre of diversity of *Uvariopsis*. Numerous other plant genera have their centre of diversity in the interval (Cheek et al., 2001) and some are endemic to it, *e.g. Medusandra* Brenan (Peridiscaceae formerly Medusandraceae, Breteler, Bakker & Jongkind (2015), Soltis et al. (2007)). The fleshy orange-red fruits of *Uvariopsis* are consumed by primates, which are thought to disperse their seeds (Dominy & Duncan, 2005). The high level of endemism of *Uvariopsis* species to the Cross-Sanaga River Interval may therefore be linked to the high number of primate species that are also endemic to the interval, bounded by the rivers Cross and Sanaga that represent barriers to primates. Ten species of primate are confined to the Interval (Kingdon, 2015).

### The range of *Uvariopsis dicaprio* and other endemic species in the Ebo Forest area

Abwe & Morgan (2008) and Cheek et al. (2018a) give overviews of habitats, species and the importance for conservation of the highly threatened Ebo Forest to which *Uvariopsis dicaprio* is restricted on current evidence. Sixty-eight globally threatened plant species are currently listed from Ebo on the IUCN Red List website (https://www.iucnredlist.org/ accessed 12 April 2021) and the number is set to rise rapidly as more of Cameroon’s rare species are assessed for their conservation status as part of the Cameroon TIPAs programme. The discovery of a new species to science at the Ebo Forest is not unusual. Numerous new plant species have been published from Ebo in recent years. Examples of other species that, like *Uvariopsis dicaprio*, appear to be strictly endemic to the Ebo area on current evidence are presented in Table 2.

**Table 2:** Plant species globally endemic on current evidence to the area of the Ebo forest, Littoral, Cameroon. Extinction risk assessment (IUCN Red List status from https://www.iucnredlist.org/ accessed 12 April 2021).

Species name	Family	Reference	Habit	IUCN status
*Ardisia ebo* Cheek	Primulaceae	Cheek & Xanthos (2012)	Herb	Critically Endangered
*Crateranthus cameroonensis* Cheek & Prance	Lecythidaceae	Prance & Jongkind (2015)	Tree	Critically Endangered
*Inversodicraea ebo* Cheek	Podostemaceae	Cheek et al. (2017)	Rheophyte	Critically Endangered
*Kupeantha ebo* M.Alvarez & Cheek	Rubiaceae	Cheek et al. (2018b)	Small tree	Critically Endangered
*Kupeantha yabassi* M.Alvarez & Cheek	Rubiaceae	Alvarez, Cheek & Sonké (2021)	Shrub	Critically Endangered (provisional)
*Palisota ebo* Cheek	Commelinaceae	Cheek et al. (2018a)	Herb	Critically Endangered
*Pseudohydrosme ebo* Cheek	Araceae	Cheek, Tchiengué & van der Burgt (2021)	Herb	Critically Endangered (provisional)
*Uvariopsis dicaprio* Cheek & Gosline	Annonacaeae	This paper	Tree	Critically Endangered (provisional)

With the exception of *Crateranthus cameroonensis* Cheek & Prance which is widespread over a large part of eastern Ebo, each of the eight species listed have on current evidence, a single small discreet range of no more than 8 km^2^ (usually far smaller) within the Ebo Forest area (see the references cited in Table 2). These species are not concentrated together in one or several spots but are scattered from the far West to the East of the forest, none of the species, apart from the *Crateranthus*, sympatric with any of the others.

Further species described from Ebo have proved not to be endemic but to have also been found further west, in the Cameroon Highlands, particularly at Mt Kupe and the Bakossi Mts (Cheek et al., 2004). Examples are *Gilbertiodendron ebo* Burgt & Mackinder, *Myrianthus fosi* Cheek (Harvey, Tchiengue & Cheek, 2010), *Salacia nigra* Cheek (Gosline, Cheek & Kami, 2014) and *Talbotiella ebo* Mackinder & Wieringa (Mackinder, Wieringa & Burgt, 2010).

Additionally, several species initially thought endemic to Mt Kupe and the Bakossi Mts and adjoining areas in the Cameroon Highlands have subsequently been found at Ebo, *e.g. Coffea montekupensis* Stoffelen et al. (1997), *Costus kupensis* Maas & H. Maas (Kamer et al., 2016), *Deinbollia oreophila* Cheek & Etuge (2009), *Microcos magnifica* Cheek (2017), and *Uvariopsis submontana* Kenfack et al. (2003). It is considered likely that additional Kupe species may yet be found at Ebo such as *Brachystephanus kupeensis* Champluvier & Darbyshire (2009), and *Impatiens frithii* Cheek & Csiba (2002) since new discoveries are still frequently being made in the Ebo Forest. Therefore, it is possible that *Uvariopsis dicaprio* might yet also be found in the Cameroon highlands, *e.g*., at Mt Kupe. However, this is thought to be only a relatively small possibility given the high level of survey effort at Mt Kupe: if it occurred there, it is highly likely that it would have been recorded already since it is so spectacular when in flower that it would be difficult to overlook.

## Conclusions

Such discoveries as this new species underline the urgency for making further such discoveries while it is still possible since in all but one of the cases given above, the species have very narrow geographic ranges and/or very few individuals, and face threats to their natural habitat, putting these species at high risk of extinction.

About 2,000 new species of vascular plant have been discovered each year for the last decade or more. Until species are known to science, they cannot be assessed for their conservation status and the possibility of protecting them is reduced (Cheek et al., 2020). Documented extinctions of plant species are increasing, *e.g. Oxygyne triandra* Schltr. and *Afrothismia pachyantha* Schltr. of South West Region, Cameroon are now known to be globally extinct (Cheek & Williams, 1999; Cheek et al., 2018c; Cheek, Etuge & Williams, 2019). In some cases, species appear to be extinct even before they are known to science, such as *Vepris bali* Cheek, also from the Cross-Sanaga interval in Cameroon (Cheek, Gosline & Onana, 2018) and elsewhere, *Nepenthes maximoides* Cheek (King & Cheek, 2020). Most of the 815 Cameroonian species in the Red Data Book for the plants of Cameroon are threatened with extinction due to habitat clearance or degradation, especially of forest for small-holder and plantation agriculture following logging (Onana & Cheek, 2011). Efforts are now being made to delimit the highest priority areas in Cameroon for plant conservation as Tropical Important Plant Areas (TIPAs) using the revised IPA criteria set out in Darbyshire et al. (2017). This is intended to help avoid the global extinction of additional endemic species such as *Uvariopsis dicaprio* which will be included in the proposed Ebo Forest IPA.

With only one locality known, *Uvariopsis dicaprio* represents another narrowly endemic Cameroonian species threatened with extinction due to deforestation for oil palm plantations, small-scale agriculture, mining and logging, widespread threats posing extinction risks to plant species in Cameroon (Onana & Cheek, 2011; Cheek et al., 2018a).

## Supplemental Information

10.7717/peerj.12614/supp-1 Supplemental Information 1 Raw data source for the plant description: the sole specimen MacKinnon 51 (holotype K001381842), holotype of *Uvariopsis dicaprio* Cheek & Gosline (K).

## Additional Information and Declarations

### Field Study Permissions

The following information was supplied relating to field study approvals (*i.e*., approving body and any reference numbers): IRAD (Institute for Agronomic Research and Development)-National Herbarium of Cameroon.

### Data Availability

The following information was supplied regarding data availability: The raw data consists of the specimen material of the new species available in the Supplemental File.

### New Species Registration

The following information was supplied regarding the registration of a newly described species: *Uvariopsis dicaprio*, 77226108-1.
