# Supplementary figures and images for: Uvariopsis dicaprio (Annonaceae) a new tree species with notes on its pollination biology, and the Critically Endangered narrowly endemic plant species of the Ebo Forest, Cameroon
Source: PeerJ. 2022 Jan 6;10:e12614. doi: 10.7717/peerj.12614 (PMC8743011; doi:10.7717/peerj.12614)

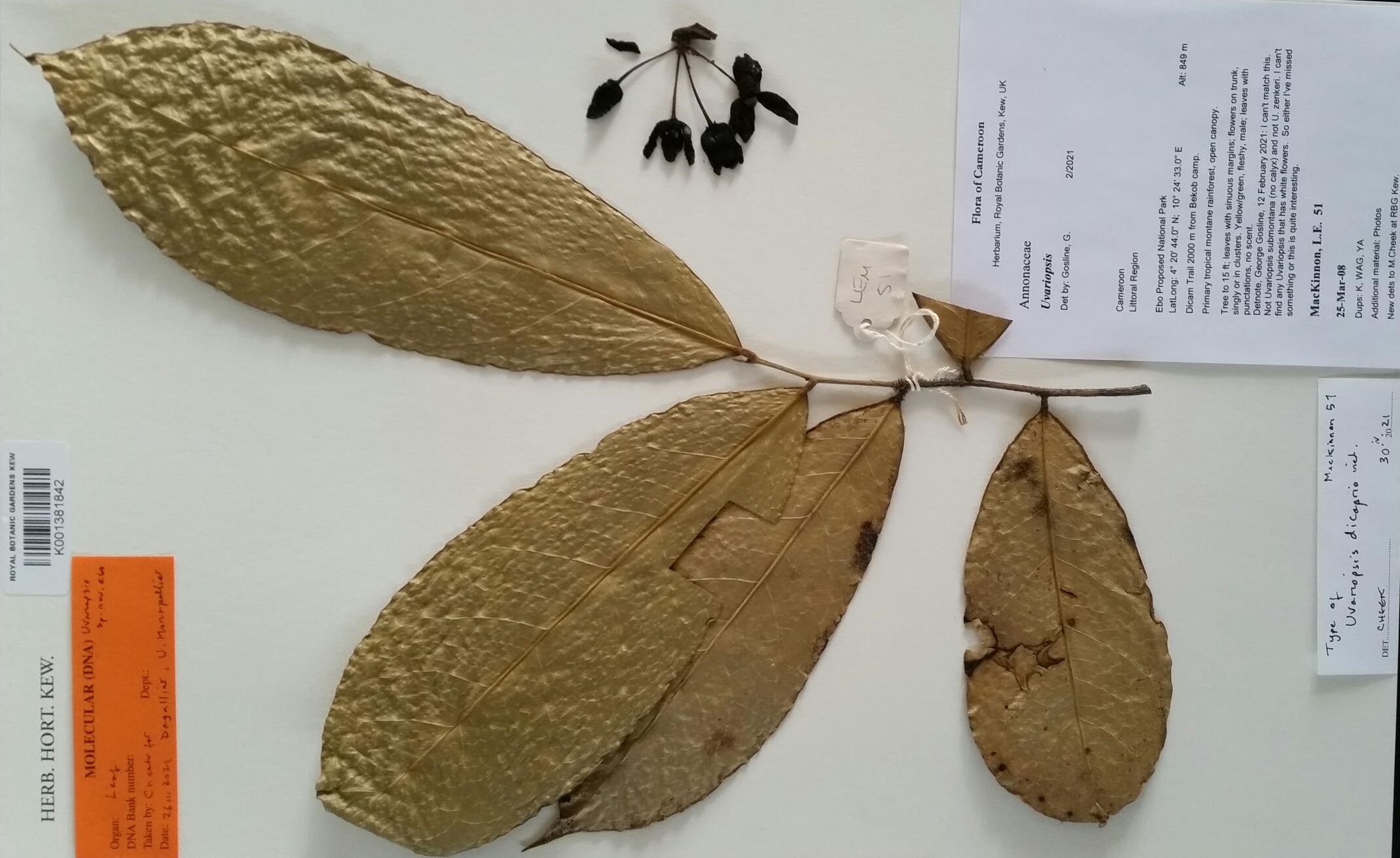

Supplement: Supplemental Information 1 [file peerj-10-12614-s001.jpg]
